# Obesity and renal cell cancer – a quantitative review

**DOI:** 10.1054/bjoc.2001.2040

**Published:** 2001-09

**Authors:** A Bergström, C-C Hsieh, P Lindblad, C-M Lu, N R Cook, A Wolk

**Affiliations:** Department of Medical Epidemiology, Karolinska Institutet, Stockholm, SE-171 77, Sweden; Department of Urology, Sundsvall Hospital, Sundsvall, SE-851 86, Sweden; Cancer Center, UMass Medical School, Worcester, MA, 01605; Department of Urology, Tian-Sheng Memorial Hospital, Pingtung, Taiwan; Department of Epidemiology, Harvard School of Public Health, Boston, MA, 02115; Division of Preventive Medicine, Department of Medicine, Brigham and Women's Hospital, Harvard Medical School, Boston, MA, 02215, USA

**Keywords:** kidney neoplasms, obesity, body weight/body mass index, risk factors, meta-analysis

## Abstract

Obesity has been associated with an increased risk of renal cell cancer among women, while the evidence for men is considered weaker. We conducted a quantitative summary analysis to evaluate the existing evidence that obesity increases the risk of renal cell cancer both among men and women. We identified all studies examining body weight in relation to kidney cancer, available in MEDLINE from 1966 to 1998. The quantitative summary analysis was limited to studies assessing obesity as body mass index (BMI, kg m^−2^), or equivalent. The risk estimates and the confidence intervals were extracted from the individual studies, and a mixed effect weighted regression model was used. We identified 22 unique studies on each sex, and the quantitative analysis included 14 studies on men and women, respectively. The summary relative risk estimate was 1.07 (95% CI 1.05–1.09) per unit of increase in BMI (corresponding to 3 kg body weight increase for a subject of average height). We found no evidence of effect modification by sex. Our quantitative summary shows that increased BMI is equally strongly associated with an increased risk of renal cell cancer among men and women. © 2001 Cancer Research Campaignhttp://www.bjcancer.com


*British Journal of Cancer* (2001) **85**(7), 984–990
© 2001 Cancer Research Campaign
doi: 10.1054/ bjoc.2001.2040, available online at http://www.idealibrary.com on IDEAL
http://www.bjcancer.com

### Obesity and renal cell cancer – a quantitative review

**A Bergström[1], C-C Hsieh[3,5], P Lindblad[1,2], C-M Lu[3,4], NR Cook[6] and A Wolk[1]**

[1]Department of Medical Epidemiology, Karolinska Institutet, SE-171 77 Stockholm, Sweden; [2]Department of Urology, Sundsvall Hospital, SE-851 86 Sundsvall, Sweden; [3]Cancer Center, UMass Medical School, Worcester, MA 01605; [4]Department of Urology, Tian-Sheng Memorial Hospital, Pingtung, Taiwan; [5]Department of Epidemiology, Harvard School of Public Health, Boston, MA 02115; [6]Division of Preventive Medicine, Department of Medicine, Brigham and Women's Hospital, Harvard Medical School, Boston, MA 02215, USA

**Summary** Obesity has been associated with an increased risk of renal cell cancer among women, while the evidence for men is considered weaker. We conducted a quantitative summary analysis to evaluate the existing evidence that obesity increases the risk of renal cell cancer both among men and women. We identified all studies examining body weight in relation to kidney cancer, available in MEDLINE from 1966 to 1998. The quantitative summary analysis was limited to studies assessing obesity as body mass index (BMI, kg $m^{-2}$), or equivalent. The risk estimates and the confidence intervals were extracted from the individual studies, and a mixed effect weighted regression model was used. We identified 22 unique studies on each sex, and the quantitative analysis included 14 studies on men and women, respectively. The summary relative risk estimate was 1.07 (95% CI 1.05–1.09) per unit of increase in BMI (corresponding to 3 kg body weight increase for a subject of average height). We found no evidence of effect modification by sex. Our quantitative summary shows that increased BMI is equally strongly associated with an increased risk of renal cell cancer among men and women. © 2001 Cancer Research Campaign http://www.bjcancer.com

**Keywords**: kidney neoplasms; obesity; body weight/body mass index; risk factors; meta-analysis

Although many epidemiological studies have shown that obesity increases the risk of renal cell cancer among women, the evidence for men is considered weaker (Wolk et al, 1996; McLaughlin and Lipworth, 2000).

The incidence of renal cell cancer, the predominant type of kidney cancer, has been increasing both in the US (Chow et al, 1999) and in most Western countries (Black et al, 1997; Liu et al, 1997). Today, renal cell cancer accounts for about 2% of cancers in the US (Landis et al, 1999), as well as worldwide (Parkin, 1998), with 30 000 cases occurring in the US in 1999 (Landis et al, 1999). The incidence varies more than 10-fold over the world. The highest rates are found in North America and Europe and the lowest in Asia (Parkin, 1998). Renal cell cancer occurs about twice as often among men, as among women.

Obesity has also been increasing throughout the world. More than half of the adult US population is considered to have an excess weight (body mass index, BMI ≥ 25.0 kg $m^{-2}$) and nearly one quarter are clinically obese (BMI ≥ 30.0 kg $m^{-2}$) (Flegal et al, 1998). The increasing prevalence of obesity might therefore, at least partly, explain the increasing incidence of renal cell cancer.

The purpose of this review was to evaluate the existing evidence that obesity increases the risk of renal cell cancer among both men and women. We conducted a quantitative summary analysis to estimate the magnitude of the association taking into account sex and different study characteristics, such as study design and size. We also investigated the possible effect modification between obesity and these factors.

*Received 2 February 2001*
*Revised 12 June 2001*
*Accepted 12 June 2001*

*Correspondence to:* A Bergström

### MATERIALS AND METHODS

### Literature review

We identified studies investigating the relation between obesity and kidney cancer, available in MEDLINE 1966 through 1998. We used the MeSH-terms 'kidney neoplasms' plus 'obesity', 'body weight' or 'body mass index' and selected original epidemiological studies. Additional studies were identified from systematical examinations of the list of references in the identified articles and previous reviews (Wolk et al, 1996; McLaughlin and Lipworth, 2000).

### Inclusion criteria

In adults, cancer of the kidney is classified as either cancer of the parenchyma (renal cell) or the renal pelvis. In many descriptive and some analytic studies tumours of the renal parenchyma and the renal pelvis are combined and the term kidney cancer is used. Since renal cell cancer is the predominant type, responsible for more than 80% of all adult kidney neoplasms (Devesa et al, 1990), studies unable to disentangle the 2 entities were included in our review.

In total, we identified 30 studies on obesity and renal cell cancer risk published between 1966 and 1998 for our review. Each study-base was eligible only once. When multiple reports were available for the same study-base we chose the one analysing incident renal cell cancer as outcome, and/or defining obesity in terms of body mass index (BMI, kg $m^{-2}$) and in more detail and/or with the greater number of cases. We excluded 6 studies (Whittemore et al, 1985; Lindblad et al, 1994; Mellemgaard et al, 1994; Moller et al, 1994; Muscat et al, 1995; Boeing et al, 1997) due to overlapping reports.

Our quantitative summary analysis was limited to studies presenting category limits of BMI and relative risk estimates with their 95% confidence interval (CI), or other results making it possible to compute these values. We excluded 9 studies (Wynder et al, 1974; Lew and Garfinkel, 1979; Whittemore et al, 1984; Yu et al, 1986; Kadamani et al, 1989; Maclure and Willett, 1990; Mellemgaard et al, 1991; Partanen et al, 1991; Finkle et al, 1993) since they did not assess obesity in a comparable way, or present their results in sufficient detail.

### Data extraction and unification

For each study we extracted the main characteristics of the study, the definition of exposure and the relative risk estimates (rate ratios, odds ratios, hereafter denoted as relative risk, RR), and their confidence intervals. All information was extracted separately for men and women. For one study, the results were not presented separately for men and women, but since crude sex-specific relative risks could be computed from the results presented in the article the study was included in the summary analysis (Talamini et al, 1990).

If the risk of renal cell cancer was expressed in more than one way, the estimate reflecting greatest degree of controlling for confounders was used. We considered age and smoking to be the most important confounding factors in the relation between obesity and renal cell cancer. We therefore grouped studies as adjusted and unadjusted depending on if they had controlled for these 2 factors.

The studies were classified as cohort or case–control, and the latter were further divided into population-based and hospital-based. Nested case–control studies, where prospectively gathered information was analysed in a case–control design, were classified as cohort studies.

The category limits in studies assessing BMI as lb $ft^{-2}$ (McLaughlin et al, 1984; Asal et al, 1988) or kg $cm^{-2}$ (Kreiger et al, 1993) were recalculated to kg $m^{-2}$. For studies assessing female obesity as kg $m^{-1.5}$ (McCredie and Stewart, 1992; Mellemgaard et al, 1995; Chow et al, 1996) we performed an approximate recalculation of these estimates to kg $m^{-2}$ by dividing the provided estimate with the square root of height. The height was set to 1.64 m, the mean height of women in recent cohort studies in Northern America, the Netherlands and Sweden (Smith-Warner et al, submitted).

### Statistical analysis

We first estimated the relative risk associated with a unit increase in BMI (1 kg $m^{-2}$) for each individual study. The odds ratio was used as a measure of the relative risk for case–control studies, and the relative risk estimates were log-transformed. When results were reported in categories of BMI, these were transformed to an estimate per unit increase in BMI. To treat BMI as a continuous exposure variable, its value was set at the midpoint of each category. For open-ended categories the approximate midpoint was set at double the distance between the midpoint and upper bound of the closest category. The log relative risk, as well as the BMI, for the reference category was set to zero (corresponding to a relative risk of 1). We subtracted the midpoint BMI of this category from the midpoint BMI of all other categories. A weighted regression was then fit through the origin where the exposure was at the reference level with log relative risk zero. The regression was weighted by the inverse variance of the log relative risk for each category. The correlation between categories was estimated using a previous method (Greenland and Longnecker, 1992).

We used a mixed effects weighted regression model to combine estimates from BMI categories from the individual studies. Between-study variation was modelled as a random effect, and heterogeneity over studies was assessed by the significance of the between-study variance (Berkey et al, 1995; Takkouche et al, 1999). The within-study variance was taken to be the estimated variance of the log relative risks for each study, giving more precise estimates greater weights in the summary measure (Greenland, 1998). Since each study contributed more than one relative risk estimate (from different BMI categories), this was a covariance matrix of dimension equal to the number of categories minus the reference. For the summary measure, we thus estimated the effect of BMI under the random-effects model while accounting for within-study correlation. To examine whether the mean of the random coefficients for BMI was the same for different groups defined by study characteristics (e.g., males versus females, US studies versus non-US studies), models including interaction term(s) between BMI and the study characteristics were further fitted and the significance of the interaction term(s) tested. The PROC MIXED procedure in SAS was applied in our analysis (SAS Institute, 1996) with the *parms* option allowing the input of known within-study variances. To examine whether a curvilinear model fits the data better than the linear, we added a quadratic term for BMI to the model.

Potential influence that unpublished data could have on our summary analysis was examined using a distortion analysis (Rosenthal, 1979). It was assumed that some studies had data on BMI and renal cell cancer risk but that the results were not published because they were not significant. To be conservative, it was assumed that the association between BMI and renal cell carcinoma in these studies was inverse. An average relative risk of 0.85 (95% confidence interval 0.67–1.08) associated with each unit increase in BMI was used. Confidence intervals including 1.0 were chosen, assuming that reports with significant associations would probably have been published.

Population attributable risk percent for renal cell cancer was estimated for persons with excess weight (BMI ≥ 25 kg $m^{-2}$) compared to those of normal weight (BMI 20–24.9 kg $m^{-2}$). We calculated the population attributable risk percent as:

$$\frac{p_e (RR-1) \times 100}{p_e (RR-1) + 1}$$

where $p_e$ represents the prevalence in the population and $RR$ represents the relative risk (Cole and MacMahon, 1971). Prevalence data were obtained from a national US survey that assessed the prevalence of excess weight in categories of overweight (BMI 25–29.9 kg $m^{-2}$), class I obesity (BMI 30–34.9 kg $m^{-2}$), class II obesity (BMI 35–39.9 kg $m^{-2}$), or class III obesity (BMI ≥ 40 kg $m^{-2}$) (Flegal et al, 1998). In this survey, 39.4% among the men were overweight, 14.6% had class I obesity, 3.6% had class II obesity, and 1.8% had class III obesity. Among women, 24.7% were overweight, 14.2% had class I obesity, 6.8% had class II obesity, and 3.9% had class III obesity. The population attributable risk percent was calculated for each category of excess weight, using the relative risk from the summary analysis, and thereafter summarized separately for each sex.

### RESULTS

### Qualitative review of the literature

In total, 24 studies on obesity and renal cell cancer with unique study bases, published between 1966 and 1998 were identified for

© 2001 Cancer Research Campaign

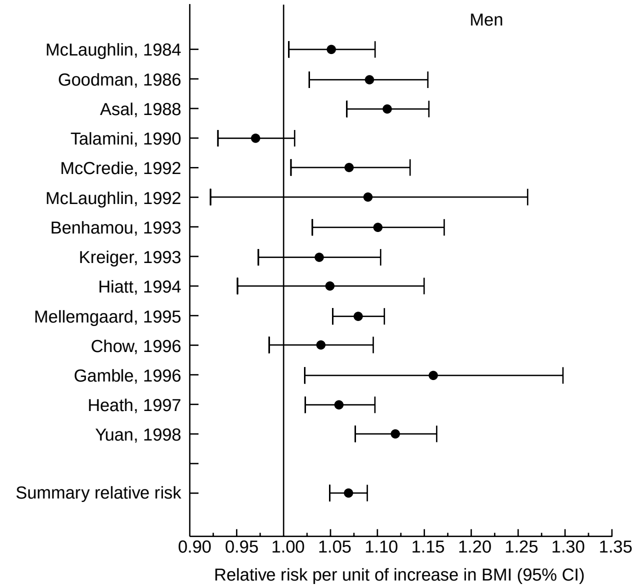

**Figure 1** Results of the reanalyses and summary analysis of published studies on the association between body mass index (BMI) and renal cell cancer risk among men. Relative risk per unit of increase in BMI (1 kg $m^{-2}$) and 95% confidence intervals (CI)

this review. Of these, 22 presented results for men and equally many for women. The individual studies are summarized in Appendix 1.

The majority of the included studies were population-based case–control studies based on incident cases of renal cell cancer, and assessed obesity as BMI or equivalent. About a third of the studies adjusted for confounding by age and smoking. In most studies the majority of the case subjects were 50 years and older, but Yu and co-workers (1986) limited their study to younger cases (15–59 years old) and in the study by Hiatt and co-workers (1994) the mean age was 50.7 years among case subjects.

An increased risk of renal cell cancer among obese men was supported by all but 2 studies. Wynder and co-workers (1974) found no effect of obesity among men, while a decreased risk was indicated in the study by Talamini and co-workers (1990). Among women, all but one study supported a positive association between obesity and renal cell cancer. In contrast, a decreased risk was indicated in the study by Talamini and co-workers (1990).

### Quantitative summary analysis

The quantitative summary analysis was based on 14 studies on each sex. The relative risks and their 95% confidence intervals for BMI as a continuous variable in studies on men are shown in Figure 1, together with the summary estimate based on a random effects model. Of the 14 studies included in the summary analysis, all but one (Talamini et al, 1990) indicated a positive association with obesity, significant in all but 4 studies (McLaughlin et al, 1992; Kreiger et al, 1993; Hiatt et al, 1994; Chow et al, 1996). The summary relative risk for all male studies combined with a random effect model was 1.07 (95% CI 1.04–1.09) per unit of increase in BMI (1 kg $m^{-2}$) (Table 1). There was some heterogeneity across studies (*P* value for between-study variance = 0.08).

The point estimates and their 95% confidence intervals for BMI as a continuous variable in studies on women are shown in Figure 2. Only one of the 14 studies included in the summary analysis showed no association between BMI and risk of renal cell cancer (Talamini et al, 1990), while all the others indicated an increased risk among obese women. This positive association was significant in half of the studies (McLaughlin et al, 1984; Kreiger et al, 1993; Mellemgaard

**Table 1** Results of summary analysis. Relative risk (RR) of renal cell cancer per unit of increase of BMI (1 kg $m^{-2}$) and 95% confidence intervals (CI)

| Group of studies | No. of studies | RR | 95% CI | Test of heterogeneity (*P* value) | Test of effect modification[a] (*P* value) |
|---|---|---|---|---|---|
| All studies | 28 | 1.07 | 1.05–1.09 | 0.03 | |
| Sex | | | | | |
| Men | 14 | 1.07 | 1.04–1.09 | 0.08 | |
| Women | 14 | 1.07 | 1.05–1.09 | 0.24 | 0.77 |
| Study design | | | | | |
| Cohort | 6 | 1.07 | 1.04–1.09 | 0.69[b] | |
| Population-based case – control | 16 | 1.08 | 1.06–1.10 | 0.23 | |
| Hospital-based case – control | 6 | 1.04 | 1.00–1.10 | 0.23 | 0.16 |
| Case status | | | | | |
| Studies with incident cases | 24 | 1.07 | 1.05–1.09 | 0.03 | |
| Studies with prevalent cases | 2 | 1.06 | 1.02–1.10 | 0.39[b] | |
| Studies with dead cases | 2 | 1.07 | 1.04–1.10 | 0.52[b] | 0.99 |
| Study size | | | | | |
| Small[c] | 12 | 1.05 | 1.02–1.09 | 0.14 | |
| Large[c] | 16 | 1.08 | 1.06–1.09 | 0.26 | 0.14 |
| Study location | | | | | |
| The US | 16 | 1.08 | 1.06–1.10 | 0.27 | |
| Countries other than the US | 12 | 1.06 | 1.03–1.09 | 0.11 | 0.24 |
| Adjusted for smoking | | | | | |
| Yes | 14 | 1.08 | 1.06–1.10 | 0.49[b] | |
| No | 14 | 1.06 | 1.03–1.09 | 0.06 | 0.32 |

[a]Possible effect modification was tested between BMI and sex, study design, case status, study size, study location, and adjustment for smoking.
[b]From fixed effect model, as within-study variance was larger than between-study variance, *P* value under mixed-effect model was 1.0.
[c]Among men: Small = studies with less than 200 cases; large = studies with 200 or more cases. Among women: Small = studies with less than 100 cases; large = studies with 100 or more cases.

 *© 2001 Cancer Research Campaign*

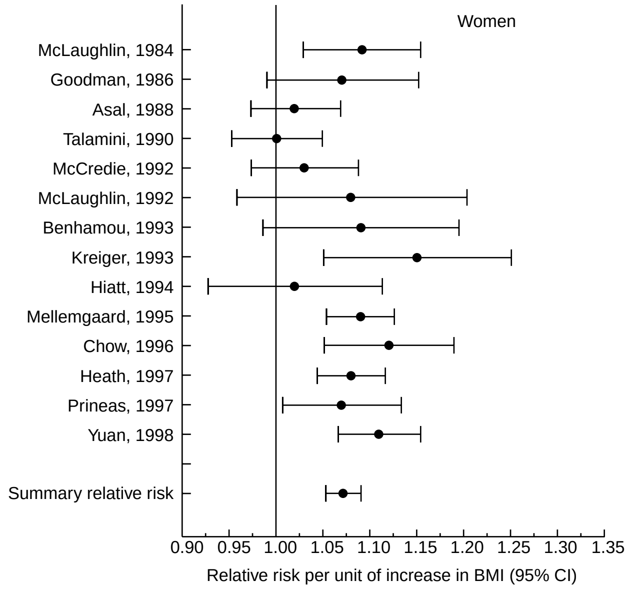

**Figure 2** Results of the reanalyses and summary analysis of published studies on the association between body mass index (BMI) and renal cell cancer risk among women. Relative risk per unit of increase in BMI (1 kg $m^{-2}$) and 95% confidence intervals (CI)

et al, 1995; Chow et al, 1996; Health et al, 1997; Prineas et al, 1997; Yuan et al, 1998). When all studies on women were combined, the summary estimate with a random effect model was 1.07 (95% CI 1.05–1.09) per unit of increase in BMI (Table 1). There was no significant between-study variance ($P = 0.24$).

The summary relative risk for men and women together was 1.07 (95% CI 1.05–1.09) per unit of increase in BMI (Table 1). One unit of increase in BMI corresponds to 3.1 kg for a man of an average height (1.77 m) and to 2.7 kg for a woman (1.64 m). When combining all studies the test for heterogeneity became statistical significant ($P = 0.03$).

We have examined whether a curvilinear model improves the fit by adding a quadratic term for BMI to the model. The quadratic term was not statistically significant in the analysis for males ($P = 0.13$), for females ($P = 0.60$), nor for all studies together ($P = 0.45$).

We hypothesized that sex, study design, case status, study size, study location or degree of adjustment for confounding might explain inter-study variability in results. However, positive associations of comparable strengths were present in all subsets of studies, regardless of study design, study size, study location (studies from the US vs. studies from other countries) or degree of adjustment for confounding (studies not adjusted for smoking vs. studies adjusted for smoking) (Table 1). For most subsets of studies, the stratum-specific between-study variance became non-significant, indicating no significant heterogeneity between the studies in these subgroups. Significant heterogeneity between studies was found only for studies with incident cases. However, some between-study variance was found for studies on men and studies not adjusted for smoking (Table 1). Furthermore, we found no effect modification between BMI and sex, study location, study design or study size, respectively (Table 1).

The potential influence that unpublished data could have on our summary analysis was examined. The distortion ('file drawer') analysis (Rosenthal, 1979) showed that if we could find 47 or more unpublished studies showing an inverse association between BMI and renal cell carcinoma, the hypothetical pooled estimate of the relative risk would no longer be significant. This indicates that our summary risk estimate is robust, and is not sensitive to potential publication bias.

The population attributable risk percent for excess weight was calculated using the prevalence in the US (Flegal et al, 1998), and the obtained relative risk of 1.07 per 1 kg $m^{-2}$ increase in BMI (corresponding to a relative risk of 1.35 for overweight subjects, 1.70 for subjects with class I obesity, 2.05 for subjects with class II obesity, and 2.40 for subjects with class III obesity compared to subjects with normal weight). We estimated that 27% of the renal cell cancer cases among American men and 29% among women could be related to overweight and obesity (BMI > 25 kg $m^{-2}$).

### DISCUSSION

In contrast to what is generally described in qualitative reviews, our quantitative summary analysis showed that the association between obesity, assessed as increased BMI, and risk of renal cell cancer was equally strong among men and women. The differences in strength of association observed across the individual studies, could not be explained by either sex, study design, case status, study size, study location or degree of adjustment for confounding.

The discrepancy between our results and previous findings, reporting stronger association among women, could be explained by the differences in distribution of BMI among men and women. Although an equal amount of men and women is overweight and obese, women tend to be more obese. In the US, where 59.4% of the men and 49.6% of the women have a BMI of 25 kg $m^{-2}$ or more, 39.4% of the men are overweight (BMI 25–29.9 kg $m^{-2}$) and 20% obese (BMI > 30 kg $m^{-2}$), while 24.7% of the women are overweight and 24.9% are obese (Flegal et al, 1998). Therefore, the highest BMI quartile for women usually consists of more obese individuals than the highest quartile for men, resulting in a higher observed relative risk.

The strengths of our review include the large number of included studies and, more important, the quantitative summary analysis comparing the relative risk of renal cell cancer is based on the same increase in BMI for both men and women.

The main limitation of our summary analysis concerns the possibility that the included studies are a biased sample of studies in general, since small studies and studies finding no association are more likely to be unpublished (Greenland, 1998). However, in our stratified summary analysis, we observed positive association of comparable strengths among small and large studies. Another concern is that not all published studies provided results that could be included in the summary analysis. In our analysis, 9 of the identified studies (Wynder et al, 1974; Lew and Garfinkel, 1979; Whittemore et al, 1984; Yu et al, 1986; Kadamani et al, 1989; Maclure and Willett, 1990; Partanen et al, 1991; Mellemgaard et al, 1991; Finkle et al, 1993) could not be included due to insufficient presented data. However, this is not likely to bias the observed association, since all but one of these studies (Wynder et al, 1974) indicated an increased risk of renal cell cancer among obese persons. The distortion analysis, used to evaluate the sensitivity of our estimates to publication bias, indicated that a large number of non-significant unpublished studies would be necessary to nullify the summary results.

Another potential limitation of our findings is that the majority of the studies in the summary analysis used retrospective

© 2001 Cancer Research Campaign self-reports of weight and height. Although such data have been shown to be quite accurate, obese subjects in general under-report their weight more than non-obese subjects while underweight subjects overestimate their body size (Kuskowska-Wolk et al, 1989). This might lead to nondifferential misclassification which, if anything, only underestimates the true association between obesity and renal cell cancer and therefore can not explain our finding of a positive association (Rothman and Greenland, 1998). We can not exclude the possibility of differential misclassification (recall bias) in the retrospective case–control studies (i.e. case subjects might report their weight differently than control subjects), but the consistency of findings from the case–control studies and the prospective cohort studies is a strong argument against recall bias.

The studies included in the summary analysis controlled for a varying degree of confounders. We considered age and smoking to be the most important confounders. All studies adjusted for age. Studies controlling also for smoking revealed a slightly higher summary relative risk than other studies. Since cigarette smoking is consistently associated with an increased risk of renal cell cancer (McLaughlin et al, 1995a) as well as with lower body weight (Williamson et al, 1991) this effect is expected. Although hypertension and diabetes (McLaughlin et al, 1995b; Schlehofer et al, 1996; Yuan et al, 1998; Lindblad et al, 1999) are possible confounders of the association between obesity and renal cell cancer, only 3 studies among men (Hiatt et al, 1994; Chow et al, 1996; Gamble et al, 1996) and 2 studies among women (Hiatt et al, 1994; Chow et al, 1996) adjusted for hypertension and none adjusted for diabetes. However, since both diabetes and hypertension could be intermediate steps in the causal pathway, it is not clear if adjustment is desirable.

Obesity might be associated with increased risk of renal cell cancer through several hormonal mechanisms. Increasing BMI is accompanied by elevated levels of fasting serum and free insulin-like growth factor-I (IGF-I) among both men and women (Frystyk et al, 1995). Insulin and IGF-I could both contribute to the growth and proliferation of renal cell cancer (Kellerer et al, 1995). Epidemiological studies indicate that patients with diabetes, which is associated with higher plasma insulin levels, have an increased risk of renal cell cancer (Schlehofer et al, 1996; Lindblad et al, 1999). Furthermore, diabetes and/or hypertension, both associated with obesity (Kopelman, 2000), might be intermediate steps in the causal pathway between obesity and renal cell cancer. Obesity also affects the hormonal milieu by increasing levels of free endogenous oestrogen (Zumoff, 1988) that may affect renal cell proliferation and growth by direct endocrine receptor-mediated effects, by regulation of receptor concentrations or through paracrine growth factors. However, though potent oestrogens have been shown to induce renal tumours in animal models (Stadler and Vogelzang, 1993), there is little epidemiological evidence supporting an association of exogenous oestrogens in humans (McLaughlin and Lipworth, 2000). Obesity could also have other effects on the kidneys. Obese individuals have been reported to have higher glomerular filtration rate and renal plasma flow independent of hypertension, which may increase risk for kidney damage (Hall et al, 1994; Ribstein et al, 1995), and therefore make the kidney more susceptible to carcinogens.

To summarize, excess body weight was positively associated with risk of renal cell cancer, equally strongly among both men and women. This finding is of great public health relevance, since it suggests that renal cell cancer can be prevented if a healthy body weight is maintained lifelong. According to our estimates, 27% of the renal-cell cancer cases among American men, and 29% among women could be attributed to overweight and obesity. This corresponds to more than 8000 cases yearly in the US and is comparable to a previous estimation (Benichou et al, 1998). Maintaining a healthy body weight can therefore be an effective strategy to prevent renal cell cancer.

### REFERENCES

Asal NR, Geyer JR, Risser DR, Lee ET, Kadamani S and Cherng N (1988) Risk factors in renal cell carcinoma. II. Medical history, occupation, multivariate analysis, and conclusions. *Cancer Detect Prev* **13**: 263–279

Benhamou S, Lenfant MH, Ory-Paoletti C and Flamant R (1993) Risk factors for renal-cell carcinoma in a French case-control study. *Int J Cancer* **55**: 32–36

Benichou J, Chow WH, McLaughlin JK, Mandel JS and Fraumeni Jr. JF (1998) Population attributable risk of renal cell cancer in Minnesota. *Am J Epidemiol* **148**: 424–430

Berkey CS, Hoaglin DC, Mosteller F and Colditz GA (1995) A random-effects regression model for meta-analysis. *Stat Med* **14**: 395–411

Black RJ, Bray F, Ferlay J and Parkin DM (1997) Cancer incidence and mortality in the European Union: cancer registry data and estimates of national incidence for 1990. *Eur J Cancer* **33**: 1075–1107

Boeing H, Schlehofer B and Wahrendorf J (1997) Diet, obesity and risk for renal cell carcinoma: results from a case control-study in Germany. *Z Ernahrungswiss* **36**: 3–11

Chow WH, McLaughlin JK, Mandel JS, Wacholder S, Niwa S and Fraumeni Jr. JF (1996) Obesity and risk of renal cell cancer. *Cancer Epidemiol Biomarkers Prev* **5**: 17–21

Chow WH, Devesa SS, Warren JL and Fraumeni Jr. JF (1999) Rising incidence of renal cell cancer in the United States. *JAMA* **281**: 1628–1631

Cole P and MacMahon B (1971) Attributable risk percent in case-control studies. *Br J Prev Soc Med* **25**: 242–244

Devesa SS, Silverman DT, McLaughlin JK, Brown CC, Connelly RR and Fraumeni Jr. JF (1990) Comparison of the descriptive epidemiology of urinary tract cancers. *Cancer Causes Control* **1**: 133–141

Finkle WD, McLaughlin JK, Rasgon SA, Yeoh HH and Low JE (1993) Increased risk of renal cell cancer among women using diuretics in the United States. *Cancer Causes Control* **4**: 555–558

Flegal KM, Carroll MD, Kuczmarski RJ and Johnson CL (1998) Overweight and obesity in the United States: prevalence and trends, 1960–1994. *Int J Obes Relat Metab Disord* **22**: 39–47

Frystyk J, Vestbo E, Skjaerbaek C, Mogensen CE and Orskov H (1995) Free insulin-like growth factors in human obesity. *Metabolism* **44**: 37–44

Gamble JF, Pearlman ED and Nicolich MJ (1996) A nested case-control study of kidney cancer among refinery/petrochemical workers. *Environ Health Perspect* **104**: 642–650

Goodman MT, Morgenstern H and Wynder EL (1986) A case-control study of factors affecting the development of renal cell cancer. *Am J Epidemiol* **124**: 926–941

Greenland S (1998) Meta-analysis. In Modern Epidemiology, Rothman K, Greenland S (ed). Lippincott-Raven: Philadelphia. 643–673

Greenland S and Longnecker MP (1992) Methods for trend estimation from summarized dose-response data, with applications to meta-analysis. *Am J Epidemiol* **135**: 1301–1309

Hall JE and Louis K, Dahl Memorial Lecture (1994) Renal and cardiovascular mechanisms of hypertension in obesity. *Hypertension* **23**: 381–394

Heath Jr. CW, Lally CA, Calle EE, McLaughlin JK and Thun MJ (1997) Hypertension, diuretics, and antihypertensive medications as possible risk factors for renal cell cancer. *Am J Epidemiol* **145**: 607–613

Hiatt RA, Tolan K and Quesenberry Jr. CP (1994) Renal cell carcinoma and thiazide use: a historical, case-control study (California, USA). *Cancer Causes Control* **5**: 319–325

Kadamani S, Asal NR and Nelson RY (1989) Occupational hydrocarbon exposure and risk of renal cell carcinoma. *Am J Ind Med* **15**: 131–141

Kellerer M, von Eye Corleta H, Muhlhofer A, Capp E, Mosthaf L, Bock S, Petrides PE and Haring HU (1995) Insulin- and insulin-like growth-factor-I receptor tyrosine-kinase activities in human renal carcinoma. *Int J Cancer* **62**: 501–507

Kreiger N, Marrett LD, Dodds L, Hilditch S and Darlington GA (1993) Risk factors for renal cell carcinoma: results of a population-based case-control study. *Cancer Causes Control* **4**: 101–110

 © 2001 Cancer Research Campaign

Kopelman PG (2000) Obesity as a medical problem. *Nature* **404**: 635–643
Kuskowska-Wolk A, Karlsson P, Stolt M and Rossner S (1989) The predictive validity of body mass index based on self-reported weight and height. *Int J Obes* **13**: 441–453
Landis SH, Murray T, Bolden S and Wingo PA (1999) Cancer statistics, 1999. *CA Cancer J Clin* **49**: 8–31
Lew EA and Garfinkel L (1979) Variations in mortality by weight among 750 000 men and women. *J Chronic Dis* **32**: 563–576
Li JJ, Li SA, Oberley TD and Parsons JA (1995) Carcinogenic activities of various steroidal and nonsteroidal estrogens in the hamster kidney: relation to hormonal activity and cell proliferation. *Cancer Res* **55**: 4347–4351
Lindblad P, Wolk A, Bergstrom R, Persson I and Adami HO (1994) The role of obesity and weight fluctuations in the etiology of renal cell cancer: a population-based case-control study. *Cancer Epidemiol Biomarkers Prev* **3**: 631–639
Lindblad P, Chow WH, Chan J, Bergstrom A, Wolk A, Gridley G, McLaughlin JK, Nyren O and Adami HO (1999) The role of diabetes mellitus in the aetiology of renal cell cancer. *Diabetologia* **42**: 107–112
Liu S, Semenciw R, Morrison H, Schanzer D and Mao Y (1997) Kidney cancer in Canada: the rapidly increasing incidence of adenocarcinoma in adults and seniors. *Can J Public Health* **88**: 99–104
Maclure M and Willett W (1990) A case-control study of diet and risk of renal adenocarcinoma. *Epidemiology* **1**: 430–440
McCredie M and Stewart JH (1992) Risk factors for kidney cancer in New South Wales, Australia. II. Urologic disease, hypertension, obesity, and hormonal factors. *Cancer Causes Control* **3**: 323–331
McLaughlin JK and Lipworth L (2000) Epidemiologic aspects of renal cell cancer. *Semin Oncol* **27**: 115–123
McLaughlin JK, Mandel JS, Blot WJ, Schuman LM, Mehl ES and Fraumeni Jr. JF (1984) A population–based case–control study of renal cell carcinoma. *J Natl Cancer Inst* **72**: 275–284
McLaughlin JK, Gao YT, Gao RN, Zheng W, Ji BT, Blot WJ and Fraumeni Jr. JF (1992) Risk factors for renal-cell cancer in Shanghai, China. *Int J Cancer* **52**: 562–565
McLaughlin JK, Lindblad P, Mellemgaard A, McCredie M, Mandel JS, Schlehofer B, Pommer W and Adami HO (1995a) International renal-cell cancer study. I. Tobacco use. *Int J Cancer* **60**: 194–198
McLaughlin JK, Chow WH, Mandel JS, Mellemgaard A, McCredie M, Lindblad P, Schlehofer B, Pommer W, Niwa S and Adami HO (1995b) International renal-cell cancer study. VIII. Role of diuretics, other anti-hypertensive medications and hypertension. *Int J Cancer* **63**: 216–221
Mellemgaard A, Moller H, Olsen JH and Jensen OM (1991) Increased risk of renal cell carcinoma among obese women. *J Natl Cancer Inst* **83**: 1581–1582
Mellemgaard A, Engholm G, McLaughlin JK and Olsen JH (1994) Risk factors for renal-cell carcinoma in Denmark. III. Role of weight, physical activity and reproductive factors. *Int J Cancer* **56**: 66–71
Mellemgaard A, Lindblad P, Schlehofer B, Bergstrom R, Mandel JS, McCredie M, McLaughlin JK, Niwa S, Odaka N and Pommer W (1995) International renal-cell cancer study. III. Role of weight, height, physical activity, and use of amphetamines. *Int J Cancer* **60**: 350–354
Moller H, Mellemgaard A, Lindvig K and Olsen JH (1994) Obesity and cancer risk: a Danish record-linkage study. *Eur J Cancer* **30**: 344–350
Muscat JE, Hoffmann D and Wynder EL (1995) The epidemiology of renal cell carcinoma. A second look. *Cancer* **75**: 2552–2557
Parkin DM (1998) The global burden of cancer. *Semin Cancer Biol* **8**: 219–235
Partanen T, Heikkila P, Hernberg S, Kauppinen T, Moneta G and Ojajarvi A (1991) Renal cell cancer and occupational exposure to chemical agents. *Scand J Work Environ Health* **17**: 231–239
Prineas RJ, Folsom AR, Zhang ZM, Sellers TA and Potter J (1997) Nutrition and other risk factors for renal cell carcinoma in postmenopausal women. *Epidemiology* **8**: 31–36
Ribstein J, du Cailar G and Mimran A (1995) Combined renal effects of overweight and hypertension. *Hypertension* **26**: 610–615
Rosenthal R (1979) The “file drawer problem” and tolerance for null results. *Psychol Bull* **86**: 638–641
Rothman K and Greenland S (1998) Precision and Validity in Epidemiologic Studies. In Modern Epidemiology, Rothman K, Greenland S (ed). Lippincott-Raven: Philadelphia. 115–134.
SAS Institute (1996) SAS/STAT software. Change and enhansment through release 6.11. SAS Institute: Cary, NC. 533–656
Schlehofer B, Pommer W, Mellemgaard A, Stewart JH, McCredie M, Niwa S, Lindblad P, Mandel JS, McLaughlin JK and Wahrendorf J (1996) International renal-cell-cancer study. VI, the role of medical and family history. *Int J Cancer* **66**: 723–726
Smith-Warner SA, Spigelman D, Yuan S-S, Adami H-O, Beeson L, van den Brandt PA, Colditz G, Folsom AR, Fraser GE, Goldbohm RA, Miller AB, Potter JD, Willett WC, Wolk A and Hunter DJ (submitted) Population Attributable Risk of Postmenopausal Breast Cancer due to Six Breast Cancer Risk Factors.
Stadler W and Vogelzang NJ (1993) Human renal cancer carcinogenesis: a review of recent advances. *Ann Oncol* **4**: 451–462
Takkouche B, Cadarso-Suarez C and Spiegelman D (1999) Evaluation of old and new tests of heterogeneity in epidemiologic meta-analysis. *Am J Epidemiol* **150**: 206–215
Talamini R, Baron AE, Barra S, Bidoli E, La Vecchia C, Negri E, Serraino D and Franceschi S (1990) A case-control study of risk factor for renal cell cancer in northern Italy. *Cancer Causes Control* **1**: 125–131
Whittemore AS, Paffenbarger Jr. RS, Anderson K and Lee JE (1984) Early precursors of urogenital cancers in former college men. *J Urol* **132**: 1256–1261
Whittemore AS, Paffenbarger Jr. RS, Anderson K and Lee JE (1985) Early precursors of site-specific cancers in college men and women. *J Natl Cancer Inst* **74**: 43–51
Williamson DF, Madans J, Anda RF, Kleinman JC, Giovino GA and Byers T (1991) Smoking cessation and severity of weight gain in a national cohort. *N Engl J Med* **324**: 739–745
Wolk A, Lindblad P and Adami HO (1996) Nutrition and renal cell cancer. *Cancer Causes Control* **7**: 5–18
Wynder EL, Mabuchi K and Whitmore Jr. WF (1974) Epidemiology of adenocarcinoma of the kidney. *J Natl Cancer Inst* **53**: 1619–1634
Yu MC, Mack TM, Hanisch R, Cicioni C and Henderson BE (1986) Cigarette smoking, obesity, diuretic use, and coffee consumption as risk factors for renal cell carcinoma. *J Natl Cancer Inst* **77**: 351–356
Yuan JM, Castelao JE, Gago-Dominguez M, Ross RK and Yu MC (1998) Hypertension, obesity and their medications in relation to renal cell carcinoma. *Br J Cancer* **77**: 1508–1513
Zumoff B (1988) Hormonal abnormalities in obesity. *Acta Med Scand* **723**(*Suppl* ): 153–160

*© 2001 Cancer Research Campaign*

**Appendix 1** Summary of published studies of obesity and risk of renal cell cancer[a]

| First author, year of publication | Type of study[b] | Country | Number of cases[c] | Exposure[d] | Categories[e] | RR | 95% CI |
|---|---|---|---|---|---|---|---|
| Wynder, 1974 | CCH | USA | 129 M | RBW[1] | No association | – | – |
| | | | 73 F | | P<0.005 for RBW 125+ | – | – |
| Lew, 1979 | Coh | USA | Unknown | RBW[2] | M: 130–139 vs 90–109 | 1.51 | – |
| | | | | | F: 140+ vs <80 | 2.03 | – |
| McLaughlin, 1984 | CCP | USA | 313 M | BMI | >28.0 vs ≤23.6 | 1.5 | 1.0–2.4 |
| | | | 182 F | | >26.2 vs ≤21.6 | 2.1 | 1.2–3.9 |
| Whittemore, 1984 | Coh | USA | 77 M | Weight (lb) | 180+ vs <140 | 2.5 | 0.9–6.8 |
| Goodman, 1986 | CCH | USA | 189 M | BMI | 28+ vs <24 | 2.67 | 1.49–5.94 |
| | | | 78 F | | 28+ vs <24 | 2.38 | 1.15–6.85 |
| Yu, 1986 | CCP | USA | 109 M | BMI | Highest vs lowest quartile | 1.8 | 0.8–4.0 |
| | | | 51 F | | Highest vs lowest quartile | 2.7 | 0.8–9.3 |
| Asal, 1988 | CCP | USA | 209 M | BMI | >29.4 vs <23.8 | 3.3 | 1.8–6.1 |
| | | | 106 F | | >30.1 vs <22.5 | 11.2 | 0.6–2.6 |
| Kadamani, 1989[f] | CCP | USA | 142 M | % standard | 140+ vs <120 | 3.8 | – |
| | | | 68 F | BMI | 140+ vs <120 | 3.0 | – |
| Maclure, 1990[g] | CCP | USA | 135 M | BMI | >28 vs ≤28 | 1.7 | 0.9–3.2 |
| | | | 68 F | | >28 vs ≤28 | 1.7 | 1.1–2.8 |
| Talamini, 1990 | CCH | Italy | 150 M+90 F | BMI | >27 vs <24 | 0.74 | 0.51–1.07 |
| Mellemgaard, 1991 | Coh | Denmark | 25 M | Obesity | | 1.52 | 0.05>P>0.001 |
| | | | 69 F | | | 2.67 | 2.08–3.38 |
| Partanen, 1991 | CCP | Finland | 338 M+F | Obesity | | 1.2 | 0.9–1.7 |
| McCredie, 1992 | CCP | Australia | 310 M | BMI | >25.34 vs <23.05 | 1.6 | 1.1–2.5 |
| | | | 179 F | $kg/m^{1.5}$ | >30.79 vs <27.21 | 1.3 | 0.8–2.1 |
| McLaughlin, 1992[h] | CCP | China | 90 M | BMI | >23.3 vs ≤19.7 | 1.7 | 0.5–5.7 |
| | | | 64 F | | >30.6 vs ≤24.4 | 3.3 | 0.7–15.1 |
| Benhamou, 1993 | CCH | France | 138 M | BMI | ≥27 vs ≤20 | 2.4 | 1.0–5.9 |
| | | | 58 F | | ≥27 vs ≤20 | 3.5 | 1.0–11.8 |
| Finkle, 1993 | Coh | USA | 191 F | $kg/m^{1.5}$ | Highest vs lowest quartile | 2.6 | 1.4–4.8 |
| Krieger, 1993 | CCP | Canada | 282 M | BMI | >25.1 vs ≤21.5 | 1.3 | 0.8–2.2 |
| | | | 181 F | | >23.0 vs ≤19.7 | 2.5 | 1.4–4.6 |
| Hiatt, 1994 | Coh | USA | 167 M | BMI | ≥28.3 vs <24.6 | 1.4 | 0.7–3.1 |
| | | | 90 F | | ≥27.8 vs <21.8 | 1.2 | 0.4–4.3 |
| Mellemgaard, 1995 | CCP | International | 1050 M | BMI | ≥26.6 vs <23.1 | 1.6 | 1.3–2.1 |
| | | | 682 F | $kg/m^{1.5}$ | ≥32.7 vs <27.3 | 2.0 | 1.5–2.7 |
| Chow, 1996 | CCP | USA | 274 M | BMI | ≥29.75 vs ≤23.13 | 1.3 | 0.7–2.3 |
| | | | 163 F | $kg/m^{1.5}$ | ≥36.57 vs ≤26.75 | 3.8 | 1.7–8.4 |
| Gamble, 1996 | Coh | USA | 37 M | BMI | ≤25 vs <21 | 3.29 | 0.93–11.62 |
| Heath, 1997 | Coh | USA | 212 M | BMI | ≥31.1 vs 20.7–24.6 | 1.6 | 0.9–2.7 |
| | | | 123 F | | ≥32.3 vs 19.1–21.9 | 3.1 | 1.5–6.4 |
| Prineas, 1997 | Coh | USA | 62 F | BMI | >28.3 vs <24.3 | 2.77 | 1.34–5.70 |
| Yuan, 1998 | CCP | USA | 781 M | BMI | ≥30 vs <22 | 4.6 | 2.9–7.5 |
| | | | 423 F | | ≥30 vs <22 | 4.0 | 2.3–7.0 |

[a]The studies by Wynder et al, 1974; Whittemore et al, 1984; Gamble et al, 1996 analysed the risk of kidney cancer unspecified. [b]Coh = cohort study; CCP = Population-based case-control study; CCH = hospital-based case–control study. [c]M = males; F = females. [d]RBW[1] = actual weight/ideal weight; RBW[2] = actual weight/average weight; BMI = kg $m^{-2}$. [e]Only highest category vs. reference category presented. [f]RR for men and women unexposed to hydrocarbon (21 cases). [g]Both men and women as reference. [h]Weight at age 50.

 *© 2001 Cancer Research Campaign*
